# Linking the Interatomic Exchange-Correlation Energy to Experimental *J*-Coupling Constants

**DOI:** 10.1021/acs.jpca.2c07693

**Published:** 2023-01-06

**Authors:** Ibon Alkorta, Paul L. A. Popelier

**Affiliations:** † Instituto de Química Médica (CSIC), Juan de la Cierva, 3, Madrid 28006, Spain; ‡ Department of Chemistry, University of Manchester, Manchester M13 9PL, U.K.

## Abstract

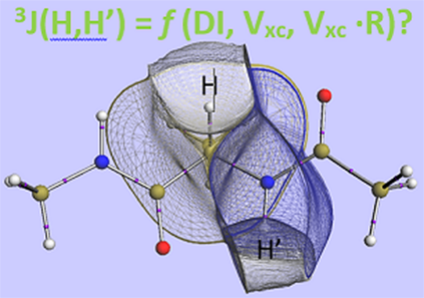

The main aim of the current work is to find an experimental connection to the interatomic exchange-correlation energy as defined by the energy decomposition method Interacting Quantum Atoms (IQA). A suitable candidate as (essentially) experimental quantity is the nuclear magnetic resonance (NMR) *J*-coupling constant denoted ^3^*J*(H,H′), which a number of previous studies showed to correlate well with QTAIM’s delocalization index (DI), which is essentially a bond order. Inspired by Karplus equations, here, we investigate correlations between ^3^*J*(H,H′) and a relevant dihedral angle in six simple initial compounds of the shape H_3_C-YH_*n*_ (Y = C, N, O, Si, P, and S), *N*-methylacetamide (as prototype of the peptide bond), and five peptide-capped amino acids (Gly, Ala, Val, Ile, and Leu) because of the protein direction of the force field FFLUX. In conclusion, except for methanol, the inter-hydrogen exchange-correlation energy V_xc_(H,H′) makes the best contact with experiment, through ^3^*J*(H,H′), when multiplied with the internuclear distance *R*_HH_′.

## 1 Introduction

Energy decomposition analysis^1^ remains a vital activity of theoretical chemistry, serving as a tool to interpret a wealth of chemical phenomena. While its roots go back about half a century,^2^ new methods continue to be added to the current plethora of energy partitioning schemes. Many of them are actually variations on older schemes in an attempt to overcome some of their shortcomings. However, a quantum topological energy partitioning called Interacting Quantum Atoms (IQA)^3^ presents a departure from the central ideas behind the more traditional but still popular energy decomposition methods. IQA is inspired by an earlier topological energy partitioning,^4^ which works for systems that are not stationary points on a potential energy surface. In contrast, the original virial partitioning,^5^ which is at the heart of the Quantum Theory of Atoms in Molecules (QTAIM),^6,7^ could only be applied to systems with vanishing forces on their nuclei. Moreover, IQA does not invoke artificial wave functions (e.g., non-Pauli) as a reference state.

IQA offers an atomistic picture of how energy is distributed, whether in a single molecule or in a molecular assembly. It provides this localized information by partitioning the total energy in intra-atomic energy contributions and interatomic ones. Besides localizing energy, IQA also delivers insight in terms of the physical type of energy that underpins any chemical phenomenon. Indeed, IQA’s interatomic energy includes the electrostatic interaction energy between any two atoms, and both the exchange energy and electron correlation energy.^8^ The intra-atomic energy consists of the atomic kinetic energy as well as the electrostatic, exchange, and correlation energy. Equipped with the capacity to provide both (atomic) location and energy type, IQA can determine the degree of covalency in a hydrogen bond, for example. Very recently, the quantum topological approach was shown^9^ to provide a real space alternative of magnetic spin coupling, one that focuses on the role of electron delocalization and one that overcomes the limitations of the orbital approach.

A question that presents itself, perhaps even imposingly, is if any of IQA’s energy contributions make contact with experiment. This question is useful in force field design, in particular, for the force field FFLUX,^10,11^ which is based on IQA. Of course, force fields are tasked to predict^12^ properties of condensed matter, for example, the self-diffusion coefficient of liquid water at various temperatures. It is clear that force fields make contact with experiments at a high level (e.g., heat capacity of constant pressure) but it would be nice to know if they also do so at a lower level (e.g., interatomic exchange energy). This may be important as a test of the soundness of the energy contributions that make up the force field. In particular, we are interested to know if the interatomic exchange-correlation energy (which is dominated by the exchange energy) can be brought in correspondence with a measurable quantity.

Interpretational quantum chemistry also benefits from the establishment of a link between the experiment and calculated atomic energy. For example, the exchange energy between two vicinal (i.e., 1,4) hydrogen atoms in staggered ethane is larger^13^ in magnitude when they are in a *trans* relationship as opposed to in a *gauche* one. This so-called “*trans* effect” (for convenience) appears counterintuitive because the hydrogens are farther away from each other while in a *trans* configuration. However, in that work,^13^ the *trans* effect was repeatedly observed in other saturated hydrocarbons, all calculated at the Hartree–Fock level of theory, which already covers exchange. It is tempting to find support for this computationally established fact from the measured *J*-coupling, also called indirect nuclear spin–spin coupling constant in full. In the case of vicinal hydrogens (protons), the relevant *J*-coupling is ^3^*J*(H,H′).

This (scalar) coupling constant quantifies the splitting of NMR resonance energies due to the coupling of nuclear spins, in this case, those of two protons separated by three bonds. Although any value of ^3^*J*(H,H′) is expressed in Hertz (Hz or s^–1^), *J*-coupling is really an energy but for a proportionality factor. In order to produce this splitting energy, *J* is multiplied by Planck’s constant (in SI units of J s) and the respective spin quantum numbers (dimensionless, e.g., 1/2 for a proton) of the two interacting nuclei. The well-known Karplus equation^14^ then offers an empirical expression, usually in the form of a heavily truncated Fourier series, that relates ^3^*J*(H,H′) to the H–A–B–H dihedral angle governing the HH interaction. In the particular case of ethane (A and B are carbon atoms), *J* reaches a local maximum when the dihedral angle is 180°, exactly when the two hydrogens are *trans* to each other. Thus, by observation, there is a potential link between the behavior of a through-space quantum topological exchange energy, V_x_(H,H′), and a measured quantity, ^3^*J*(H,H′).

Already in 1996, Bader et al. observed,^15^ within the context of QTAIM, that a measure of Fermi-hole density delocalization between two vicinal hydrogens (denoted F(H,H′)) correlates very well with the coupling constant ^3^*J*(H,H′), both following the same Karplus equation. The Fermi hole is a consequence of the Pauli exclusion principle, which states that no two electrons with the same spin can occupy the same point in space. To understand the Fermi hole, let us fix an electron of a given spin at a fixed position. There will thus be a decrease in the probability of finding another electron with the same spin relative to this given electron. The Fermi hole is a distribution function that quantifies^16^ this decrease relative to an uncorrelated pair density. As an electron moves through space, it carries with it a Fermi hole as “no go zone” of an ever-changing degree and shape. Returning to F(H,H′), it is curious that this quantity does not adopt the dimension of energy but rather that of a delocalization index (DI), which is often identified with a bond order, and which is derived from F(A,B) values. The DI represents the number of electrons exchanged or shared between two given atoms.^17^ The DI has also been used as a measure of the bond order^18−22^ and the average value between *para* atoms in six-membered rings has been used to characterize the aromaticity of these rings.^23^ Referring for the mathematical details to the mature literature,^24,25^ we just summarize here that F(H,H′) is calculated as a double sum of products over atomic overlap integrals. The latter are denoted *S_ji_*(Ω_H_)*S_ij_*(Ω_H′_) and defined in Section 2, where the indices *i* and *j* refer to spin orbitals, and Ω_A_ to the volume of atom A over which the orbitals are integrated. Being aware, at this point, of this stub of a formula is important to understand the following argument.

The observation that *^N^J*(A,B) (which is more general than ^3^*J*(H,H′)) is proportional^26^ to doubly summed products of overlap integrals can be rationalized by going back to McConnell’s formula^27^ published in 1956. His equation, which is also given and discussed in detail elsewhere,^25^ is based on the valid assumption that the dominant term (out of three terms) in the *J*-coupling Hamiltonian^28,29^ is the Fermi contact (FC) term. This term consists of the magnetic interaction between the nuclear spin and the electron spin, which is much larger than the direct interaction between two nuclear spins. The FC interaction is the magnetic interaction between an electron and an atomic nucleus when the electron is at the nucleus. This is why elaborate basis sets with excellent core-electron flexibility are important for accurate calculations of the *J*-coupling. The FC offers a mechanism^25^ to transmit the presence of the spin of one nucleus to that of another nucleus. Indeed, the spin magnetic moment of nucleus A polarizes the spin density of the electrons in its immediate neighborhood, which leads to a small excess of oppositely polarized electron spin density. This information is then transmitted to the vicinity of another nucleus B via the mechanism of electron exchange. This fact is compatible with the appearance of exchange terms (i.e., atomic overlap integrals) in McConnell’s formula. However, to be more precise, this is only true if the density at the proton parallels the increase of the density within the volume of the hydrogen atom. As a result, one can prove that *^N^J*(A,B) will depend linearly on  and thus on the DI or bond order.

Matta et al.^25^ computationally verified the validity of the last statement for ^3^*J*(H,H′) in a small series of polyaromatic compounds as well as for a handful of saturated hydrocarbons. At first glance, this result is expected, following the reasoning starting with McConnell’s equation, because the atomic volume of the hydrogen atom is primarily described by spherically symmetric *s*-functions. However, it will then come as a surprise that ^3^*J*(F,F′) also depends^30^ linearly on the respective DI for 29 fluorinated aromatics, spanning a *J*-coupling range of 85 Hz. Indeed, one would expect the approximate link with *S_ji_*(Ω_F_)*S_ij_*(Ω_F′_) to fail for non-hydrogen atoms due to the presence of basis functions with nonzero angular momentum, which increase^31^ the contribution to the *J*-coupling due to terms other than the FC term. Still, these authors phenomenologically showed that the correlation even holds with oxygen atoms intervening between the coupled fluorine atoms (although not in compounds with crowded fluorine atoms).

Finding a successful correlation between experimental *^N^J*(H,H′) (with *N* = 1,2,3,4,...) values and the corresponding DIs for heteroaromatics containing oxygen (as well as nitrogen, sulfur, and selenium) is unique to the QTAIM partitioning. Mandado et al. have shown^32^ that the alternative partitioning method of Mulliken is useless (in their words) since no correlation was found for the original set of polybenzoids of Matta et al.^25^ when extended with 5- and 6-membered heteroaromatics. The Hirshfeld partitioning scheme fares better than that of Mulliken but shows the reduced correlation for hydrogens placed in the presence of electronegative atoms. That work^32^ justifies the current study’s use of the QTAIM partitioning when studying (doubly peptide-) capped amino acids, compounds of interest to the development of FFLUX. It is worth reflecting for a moment on how an *ab initio* program is able to provide *J*-coupling, localized between two atoms, without ever invoking a partitioning method. The integrals in McConnell’s original formula are calculated over all space, yet they allow themselves to be approximated by integrals over the finite volume of quantum topological atoms. Even the atomic weight factor of Hirshfeld’s partitioning corroborates the somehow natural localization that characterizes *J* values.

Using QTAIM, more successful correlations^33^ have been obtained since the pioneering work mentioned above: B3LYP^34^ calculations on seven 9-substituted anthracene derivatives confirmed linear correlations between the DI and both vicinal (^3^*J*) as well as long-range (^4^*J*) proton–proton coupling constants, respectively. Furthermore, an MCSCF study of hydrogen dissociation in protonated benzene shows that a larger QTAIM CH delocalization index implies^35^ a larger CH *J*-coupling. Another QTAIM B3LYP study showed^36^ an accurate correlation between the HH DI and HH *J*-coupling of a highly investigated, bioactive diterpenoid. Finally, a striking similarity was found^37^ between the variation of the *J*-coupling with the intermolecular distance in noncovalent interactions and the same type of plot for the Laplacian of the electron density at the intermolecular bond critical point.

It is against the extensive background outlined above that we now investigate, for the first time to the best of our knowledge, if there is a correlation between the coupling constant ^3^*J*(H,H′) and the interatomic exchange-correlation energy V_xc_(H,H′). Although we will investigate this matter in the above phenomenological tradition of this type of calculations, we start by reviewing the established theoretical link between V_xc_(H,H′) and DI(H,H′). This link will help in interpreting the observed results.

## 2 Theoretical Background

### 2.1 Exchange Energy and Delocalization Index

The IQA method is the most used topological energy partitioning method (see ref (38) for an alternative) to divide the total electronic energy of a system into intra-atomic and interatomic contributions,
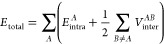
1where *A* and *B* label different (quantum topological) atoms. The interatomic energy can be further partitioned into contributions associated with nuclei (n) and electrons (e) in each atom:
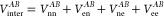
2where a vertical match between subscript and superscript tells which entities (n or e) interact. Finally, the electron–electron potential energy *V*_ee_^*AB*^ can be even further partitioned into a Coulombic, exchange, and correlation term:
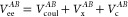
3where the (alternatively written) exchange term V_x_(A,B) provides information on the degree of covalent bonding between *A* and *B.*

As we will work with B3LYP wave functions, the interatomic exchange energy is written in terms of Kohn–Sham (KS) orbitals ψ_*i*_^KS^, by following the template of (closed-shell in our case) Hartree–Fock theory:
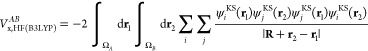
4where the KS orbitals simply replace the Hartree–Fock orbitals and the two consecutive 3D integrals constitute a 6D integral over the volumes of atom *A* and *B.* The subscript X emphasizes that, formally, we extract only the exchange part from the exchange-correlation energy. We should and will actually write V_xc_(A,B) and also mention that the total molecular energy can be sufficiently accurately recovered by the first ever DFT-IQA partitioning scheme proposed^39^ a few years ago. In eq 4, **R** = **R_B_** – **R_A_** represents the internuclear vector while **R_B_** and **R_A_** are the respective nuclear position vectors of nucleus *B* and *A.* The vectors **r_1_** and **r_2_** describe the electron density in the volumes of atom *A* and *B*, respectively. It should be noted that the magnitude of the compound vector appearing in the denominator, |**R + r_2_** – **r_1_**|, is the distance between two infinitesimal pieces of electron density, one in *A* and one in *B*. Rewriting eq 4 using the overlap function *S_ij_*(**r**) = ψ_*i*_^KS^(**r**)ψ_*j*_^KS^(**r**) leads to
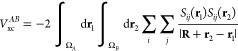
5

Following a derivation published^40^ in 2007, a Taylor expansion of |**R** + **r_2_** – **r_1_**|^–1^ and subsequent use of an addition theorem for regular spherical harmonics (*R_lm_*(**r**)) factorize the electronic (**r_1_** and **r_2_**) and nuclear coordinates (**R**) as follows:
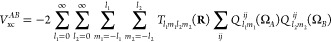
6where we call *Q_lm_^ij^*(Ω) exchange (multipole) moments in analogy with the electrostatic multipole moments *Q_lm_*(Ω), which depend on the electron density rather than a product of two orbitals; this is why they lack an imprint of the orbitals *i* and *j*. The exchange moments are defined by
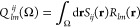
7

Equation 7 can now be truncated to the first term only, by setting *l* = *m* = 0 and using the fact that *R*_00_(**r**) = 1 and *T*_00,00_(**r**) = 1,
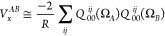
8where *R* is the internuclear distance. It is pleasing to spot the similarity between eq 8 and *q*_A_*q*_B_/*R*, which expresses the electrostatic interaction between two point charges *q*. Both equations result from a truncated multipolar expansion of an energy that actually depends on an electron density or an exchange density. For our purpose, it is important to connect eq 8 with the DI, which is defined as
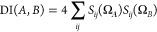
9where
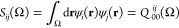
10

Substituting eq 9 into eq 8 yields the equation that is central to this paper:
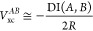
11

A study^40^ of the convergence behavior showed that this equation is quite a good approximation, unless *A* and *B* are very close, such as in a 1,2 relation, in which case the expansion typically diverges. However, as a successful example of convergence, eq 11 returns an approximate exchange energy of −4.8 kJ/mol for two hydrogen atoms interacting in a *trans* relation in acrolein, being 3.1 Å away from each other, while their exact exchange energy is −4.0 kJ/mol. However, many atoms separated by more than 3.5 to 4 Å reveal that the approximation at the heart of eq 11 holds within 0.1 kJ/mol. As a result, one expects the expression DI(A,B) = 2*R*_AB_|V_xc_(A,B)| to hold quite well, which is important to interpret the observed results.

### 2.2 *J*-Coupling Constants

Among the different relationships that correlate structural characteristics with NMR parameters, the Karplus equation is widely used. This equation relates ^3^*J*(A,B) with the value of the dihedral angle (A–X–Y–B), θ. The equation was originally developed for H–H *J*-couplings, ^3^*J*(H,H′), but later extended to other nuclei.^41−43^ The Karplus equation can be written as eq 12 or eq 13, where we use eq 12 in this work,
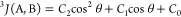
12
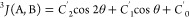
13It should be noted that Karplus published another paper^44^ four years after his original one, partially motivated by some criticism on the limitation of this theory. He analyzed the effect of the substituent’s electronegativity and the dependence on the bond angle and bond length, but the latter turned out to be of minor relevance.^43^ It is known that *J*-couplings associated with the backbones of peptides and proteins are actually functions of both the φ and ψ dihedral angles rather than simply φ or ψ, but for our purpose, this insight is not crucial. Interpolations^45^ obtained from thousands of geometries over a two-dimensional dihedral angle grid can be used to more accurately determine peptide and protein structures from NMR measurements in solution. Finally, we point out^43^ that measured *J*-couplings are actually a time average of instantaneous values over a time scale of milliseconds. In the static approach, one uses an individual structure that represents the averaged dihedral angles. The motional averaging effects are automatically included in the adjusted coefficients during the parameterization process.

## 3 Materials and Computational Details

In their assessment, against the experiment, of using DFT, CCSD, and MCSCF for the calculation of *J*-couplings in ethane, methanol, and methylamine, Pecul and Helgaker concluded^46^ that B3LYP^34^ performs well for geminal ^2^*J*(H,H′) and ^2^*J*(C,H) couplings but tends to slightly overestimate the vicinal ^3^*J*(H,H′) couplings, although B3LYP is sufficiently accurate for most purposes. Thus, we employed the B3LYP functional in combination with different basis sets in the calculation of ^3^*J*(H,H′) for ethane in order to select the most adequate basis set against the experiment. Ethane has been measured under various conditions, and hence, a range of five experimental values was reported^47^ spanning 7.992 and 8.005 Hz. The results for CH_3_CH_3_, CH_3_NH_2_, and CH_3_OH have been gathered in Table S1 of the Supporting Information (SI). The IGLO-III basis set^48^ provides the value (7.88 Hz) closest to the experiment for ethane for all eight basis sets considered, by only ∼0.1 Hz. The reliability of this basis set is confirmed by comparing the experimental and calculated values of ^3^*J*(H,H′) in CH_3_OH (Exp^49^ = 5.535 Hz, Calc = 5.82 Hz) and CH_3_NH_2_ (Exp^50^ = 7.0 Hz, Calc = 7.00 Hz). Consequently, the geometries of the energy minima of all systems were obtained at the B3LYP/IGLO-III level and a rotational energy profile was generated by changing the H–C–Y–H angle in increments of 15° and then reoptimizing the rest of the geometrical parameters. The minima of the capped glycine, alanine, valine, leucine, and isoleucine were taken from a previous study where the potential energy surface was explored for 20 natural amino acids.^51^ The ^3^*J*(H,H′) values were calculated at the B3LYP/IGLO-III level of theory. All calculations were carried out with the GAUSSIAN09 program,^52^ while DI and V_xc_ values were calculated with the AIMAll program^53^ using the B3LYP wavefunction by a method that makes IQA compatible^39^ with this functional.

**Figure 1:**
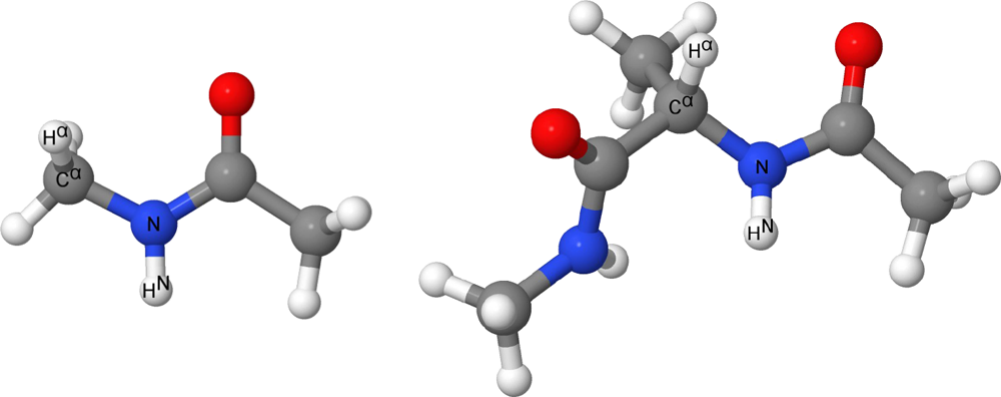
Labeling of the H^N^–N–C^α^–H^α^ dihedral angle that is varied in the calculation of ^3^*J*(H^N^,H^α^) in NMA (left) and alanine (right). It should be noted that this dihedral angle is labeled as θ throughout the paper and the SI for convenience.

We explored the correlation between ^3^*J*(H,H′) and two quantities, DI and V_xc_, between the two hydrogens of interest in a set of six simple molecules: CH_3_CH_3_, CH_3_NH_2_, CH_3_OH and their period-3 semi-counterparts, CH_3_SiH_3_, CH_3_PH_2_, and CH_3_SH, summarized as H_3_C-YH_*n*_ (where Y = C, N, O, Si, P, or S with *n* = 1, 2, or 3 depending on element *Y*). In addition, we have calculated correlations for *N*-methylacetamide (NMA), which is a prototype molecule representing the peptide bond in proteins. Increments of 15° in the H^N^–N–C^α^–H^α^ dihedral angle of NMA were used to control the scanning of its energy profile. Finally, we also calculated the correlations for five capped amino acids (Gly, Ala, Val, Leu, and Ile) in their local energy minima and along φ dihedral energy profiles^54^ centered on the global energy minimum. First, the ψ dihedral angle of the global minimum was frozen, and then, the φ dihedral angle was rotated by 15° increments between −180° and +180°, resulting in 24 = [180 – (−180)]/15 geometries, additionally to the global minimum. The 24 geometries were then relaxed through geometry optimization, keeping both the φ and ψ dihedral angles frozen. Figure 1 shows the H^N^–N–C^α^–H^α^ dihedral angle in NMA and alanine as a representative of the five capped amino acids.

## 4 Results and Discussion

The calculated ^3^*J*(H,H′) values for the H_3_C-YH_*n*_ series and NMA as well as the corresponding DI(H,H′) and V_xc_(H,H′) values have been gathered in Table S2 of the SI. The ^3^*J*(H,H′) values fit nicely to Karplus equations showing coefficients of determination, *R*^2^, with values larger than 0.98 for all the H_3_C-YH_*n*_ molecules (Table S3). In the case of the capped amino acids (glycine, alanine, valine, leucine, and isoleucine), two sets of ^3^*J*(H^N^,H^α^) values are considered. One set consists of *J*-couplings from the dihedral φ scan, centered on the global minimum for each of the five amino acids, and a second set incorporates the previous values plus those of all the reported local energy minima.^51^ For all 5 amino acids, the fitting of the dihedral scan values provides better statistics (*R*^2^ > 0.95) than when all the minima are included (*R*^2^ > 0.93). It should be noted that because ^3^*J*(H,H′) is dominated by the FC term, similar correlations are found for FC as a function of the dihedral angle. Indeed, Table S4 confirms linear relationships between the total ^3^*J*(H,H′) and the FC term, showing a slope very close to 1.0, intercepts close to 0.0, and *R*^2^ values larger than 0.998.

**Figure 2:**
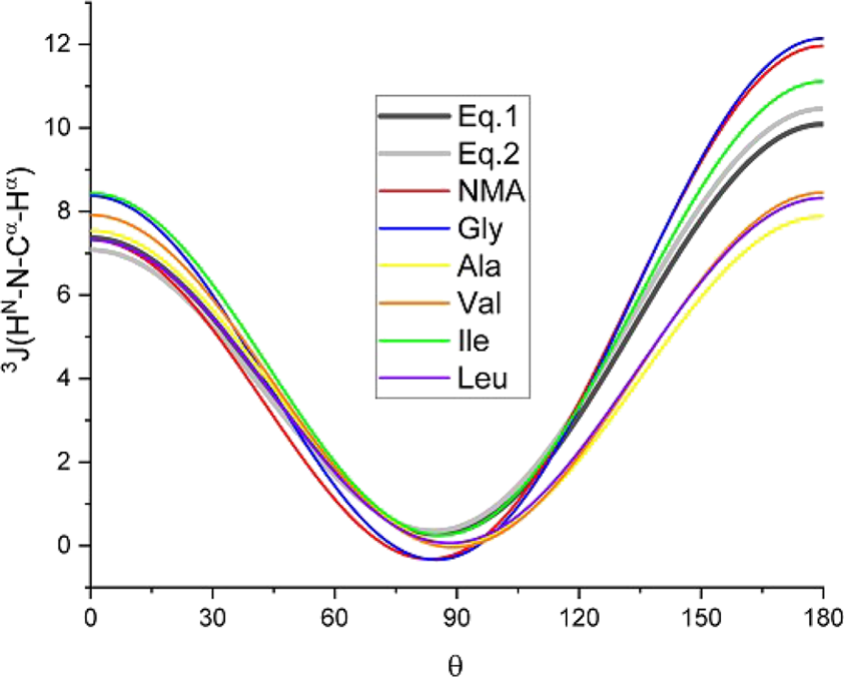
^3^*J*(H^N^,H^α^) for NMA, Gly, Ala, Val, Ile, and Leu as a function of the dihedral angle θ (specified in Figure 1), fitted to either values calculated in this work or fitted to experimental data.^43^

The calculated ^3^*J*(H^N^,H^α^) values for NMA and the five amino acids can be clustered in two groups. The first group comprises NMA, Gly, and Ile, which show values of ^3^*J* for |θ| = 180° around 12 Hz while the systems in the second group (Ala, Val, and Leu) show values around 8 Hz for the same angles. The fitted Karplus equations based on our calculated *J*-coupling values and the two Karplus equations reported based on experimental studies show a reasonable agreement, as can be concluded from Figure 2. It should be noted that this figure plots the “scan” equations listed in the second part of Table S3. The agreement is encouraging especially considering that the experimental values correspond to average values of all the amino acids and the fact that the Karplus equation is affected by the substituents^55^ and the side-chain conformation.^56^ It should be noted that what we refer to here as experimental *J*-coupling values are actually calculated from two equations (eq 1 and eq 2 in Figure 2) obtained by an ensemble fitting method applied to two different proteins, the details of which can be found in the top two entries of Table 5.7 of ref (43).

The use of a single Karplus equation to fit all the calculated *J*-coupling values for NMA and the amino acids together (259 points) provides a much smaller *R*^2^ value (0.88, Table S3) due to the scattering of values in the amino acids previously mentioned in the data set.

The correlations between ^3^*J*(H^N^,H^α^) and the corresponding FC term are excellent (*R*^2^ = 0.999) for NMA, Gly, and Ile (Table S4). However, for Ala, Val, and Leu, the *R*^2^ decreases to 0.94. However, after separating the data in two intervals of the dihedral angle (|θ| > 90° and |θ| < 90°), excellent correlations are again found. Table S4 also shows plots of Ala, Val, and Leu where the contribution of the FC term to the total ^3^*J*(H,H′) coupling constant is larger if |θ| < 90° than if |θ| > 90°.

Table 1 shows Karplus-like relationships between V_xc_ and the relevant dihedral angle θ with good correlation (*R*^2^ > 0.93) even when all the values for NMA and the amino acids are considered together (*R*^2^ = 0.937). Table S5 shows Karplus-like relationships between DI and θ with excellent correlation (*R*^2^ > 0.97) holding out very well even when all the values for NMA and the amino acids are considered together again (*R*^2^ = 0.98). In all cases, the largest ^3^*J*(H,H) and DI values are associated with a dihedral angle of 180°. For V_xc_, the largest absolute value is found at 0° for CH_3_-YH_*n*_, with Y = C, Si, P, and S and all the amino acids while it is at 180° for Y = N and O, and for NMA.

**Table 1:** Karplus-Like Relationships between V_xc_ (kJ/mol) and the Dihedral Angle, θ, which Is H^N^–N–C^α^–H^α^ or H–C–Y–H

system	fitted equation [*x* = cosθ]	*R*^2^
CH_3_-CH_3_	V_xc_ = −1.327*x*^2^ – 0.172*x* – 0.216	0.998
CH_3_-NH_2_	V_xc_ = −0.941*x*^2^ + 0.034*x* – 0.169	0.991
CH_3_-OH	V_xc_ = −0.717*x*^2^ + 0.087*x* – 0.113	0.9995
CH_3_-SiH_3_	V_xc_ = −0.9890*x*^2^ – 0.148*x* – 0.334	0.987
CH_3_-PH_2_	V_xc_ = −1.433*x*^2^ – 0.351*x* – 0.365	0.939
CH_3_-SH	V_xc_ = −1.300*x*^2^ – 0.260*x* – 0.246	0.979
NMA	V_xc_ = −0.623*x*^2^ + 0.0846*x* – 0.129	0.994
Gly (scan + min)	V_xc_ = −0.682*x*^2^ + 0.043*x* – 0.102	0.960
Ala (scan + min)	V_xc_ = −0.718*x*^2^ – 0.053*x* – 0.147	0.970
Val (scan + min)	V_xc_ = −0.796*x*^2^ – 0.043*x* – 0.089	0.969
Ile (scan + min)	V_xc_ = −0.764*x*^2^ – 0.041*x* – 0.102	0.959
Leu (scan + Min)	V_xc_ = −0.694*x*^2^ – 0.030*x* – 0.139	0.983
All-AA + NMA	V_xc_ = −0.734*x*^2^ – 0.015*x* – 0.110	0.937

**Figure 3:**
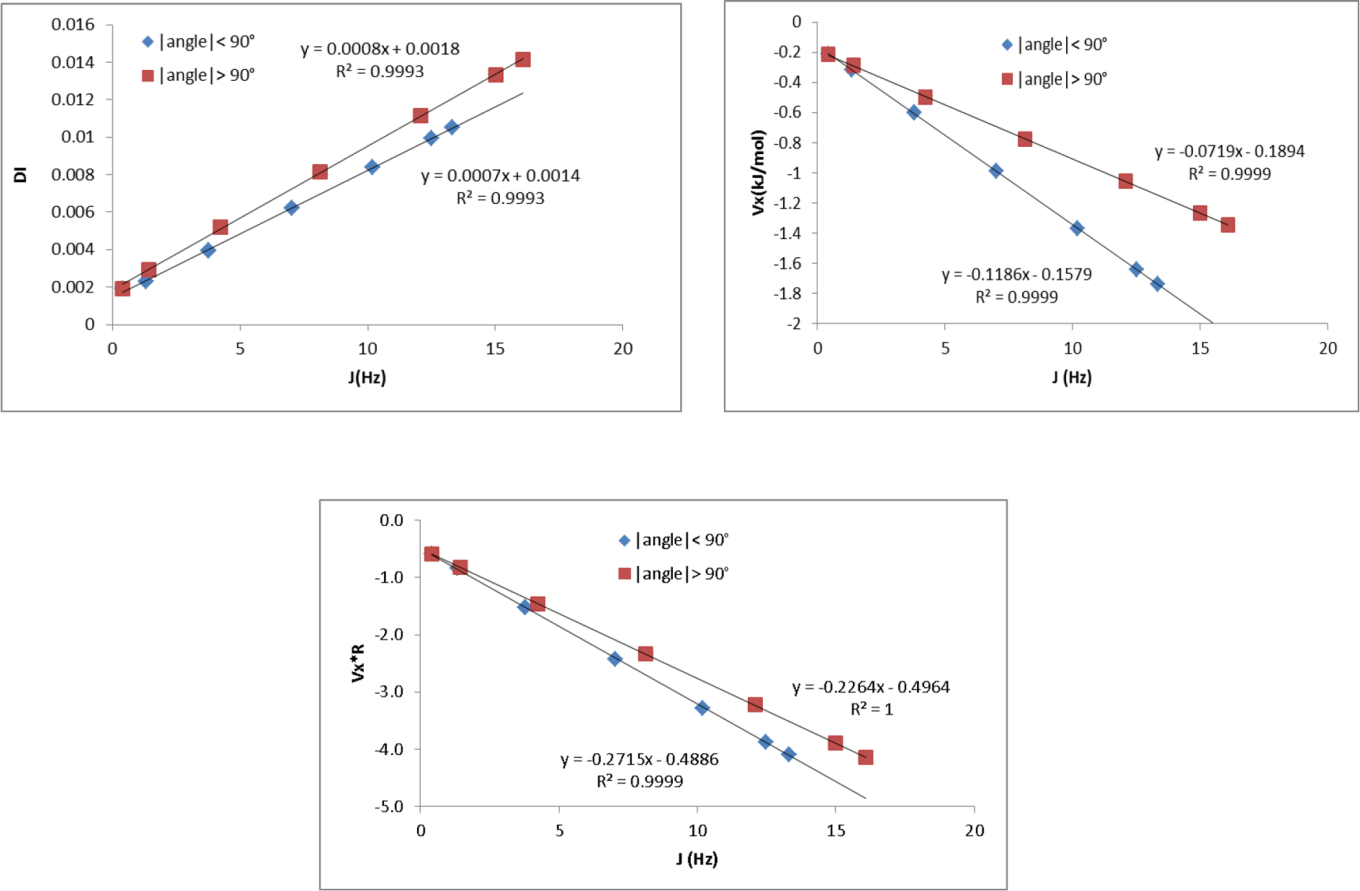
DI (top left), V_xc_ (kJ/mol) (top right), and V_xc_*R* (kJ/mol Å) (bottom) as a function of *J* (Hz) in ethane. The values are separated in two groups based on the values of the dihedral angle θ.

The linear correlation between ^3^*J*(H,H′) and DI shows good *R*^2^ values for DI (*R*^2^ > 0.9) while for V_xc_, the results are more variable, with *R*^2^ values between 0.5 and 0.99. However, the behavior of V_xc_ and DI as a function of the *J*-couplings shows that the data points are separated in two different trends depending on the value of θ. Figure 3 illustrates this effect for ethane while Figure S1 shows this for all other compounds. Those configurations with |θ| < 90° show larger values of V_xc_ and DI than those with |θ| > 90° for a given ^3^*J*(H,H) value. The two fitted lines converge (i.e., intersect) for the smaller values of ^3^*J*(H,H′), which are associated with a θ value of about 90°. The differences between the two subsets in the V_xc_ and DI parameters very much depend on the molecule, being maximal for the V_xc_ values of CH_3_-SiH_3_, while in the same molecule, the values of DI almost fully coincide (i.e., the angle between the two fitted lines collapses to nearly zero (Figure S1)).

**Figure 4:**
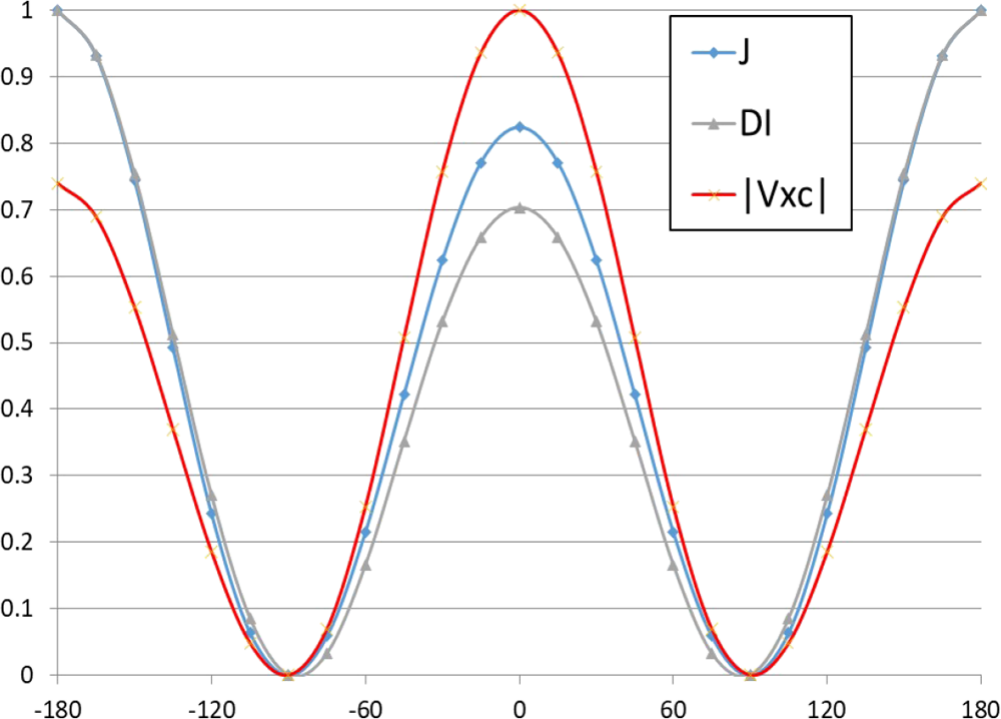
Normalized values (between 0 and 1) of ^3^*J*(H,H), DI, and −V_xc_ for ethane.

The splitting of the values in two linear correlations (Figure 3) can be understood if the normalized values (between 0 and 1) of the three properties are plotted simultaneously (see Figure 4 for ethane). The largest normalized values for *J* and DI in ethane correspond to θ = 180° (−180°). The normalized values of these two properties perfectly overlap in the range of values of |θ| > 90°. However, this is not the case for |θ| < 90°. Consequently, the same linear correlations cannot hold for both |θ| > 90° and |θ| < 90°. The absolute value of V_xc_ is the largest at θ = 0°, and for |θ| < 90°, its normalized values are larger than those of *J* while the opposite is true for |θ| > 90°. The correlation of normalized |V_xc_| versus *J* should have a slope larger than 1.0 for |θ| < 90° and one smaller than 1.0 for |θ| > 90°. Similar results are obtained when the FC term is used versus DI and V_xc_ (Figure S2).

Because, according to eq 11, the DI is approximately proportional to V_xc_*R* (where *R* is the internuclear distance), the correlation between V_xc_*R* and ^3^*J*(H,H) was checked in the whole range of angles. Table 2 allows a full comparison between fits of ^3^*J*(H,H′) against DI, V_xc_ or V_xc_*R* for all 12 compounds. The first important observation is that the fits to V_xc_*R* give (mostly substantially) higher *R*^2^ values than those to V_xc_ for all compounds except one. The exception is CH_3_OH where the correlation of V_xc_ (*R*^2^ = 0.991) barely beats that of V_xc_*R* (*R*^2^ = 0.983). Second, the correlations of V_xc_*R* with ^3^*J*(H,H) shows larger *R*^2^ values than those between DI and ^3^*J*(H,H) in 4 of the 6 small compounds. The two exceptions are CH_3_-SiH_3_ and CH_3_-PH_2_, while for CH_3_-CH_3_, the fits are almost identical (0.978 and 0.973). Thirdly and importantly, in the set of NMA with the amino acids, V_xc_*R* is the best single descriptor with *R*^2^ values between 0.95 and 0.99. The fact that fits to DI are always the worst (and sometimes markedly so) shows that the rationale offered by eq 11 cannot be taken for granted. In particular, the worst DI correlations are found for Ala, Val, and Leu, which were identified earlier in the subset for which the correlation between ^3^*J*(H^N^,H^α^) and the FC term was worse than that for the other subset of NMA, Gly, and Ile.

**Table 2:** Coefficient of Determination, *R*^2^, of the Regression of ^3^*J*(H,H′) against DI, V_xc_, and V_xc_*R*

system	DI	V_xc_	V_xc_*R*
CH_3_-CH_3_	0.978	0.828	0.973
CH_3_-NH_2_	0.916	0.965	0.993
CH_3_-OH	0.902	0.991	0.983
CH_3_-SiH_3_	0.997	0.521	0.786
CH_3_-PH_2_	0.980	0.678	0.913
CH_3_-SH	0.940	0.804	0.965
NMA	0.918	0.941	0.992
Gly	0.934	0.909	0.981
Ala	0.784	0.920	0.964
Val	0.779	0.889	0.948
Ile	0.931	0.708	0.958
Leu	0.752	0.868	0.941

## 5 Conclusions

The IQA method is a relatively new addition to energy decomposition analysis. It mandatorily operates at the atomic level because it adopts the quantum topological atom defined by the Quantum Theory of Atoms in Molecules (QTAIM). IQA is at the basis of the force field FFLUX while its various atomistic energy contributions provide chemical insight (e.g., the origin of the rotation barrier in biphenyl). The main aim of the current work is to find an experimental connection to IQA’s interatomic exchange-correlation energy V_xc_(A,B). A suitable candidate as (essentially) experimental quantity is the NMR *J*-coupling constant denoted ^3^*J*(H,H′), which a number of previous studies showed to correlate well with QTAIM’s the DI, which is essentially a bond order.

Inspired by Karplus equations, here, we investigate correlations between ^3^*J*(H,H′) and a relevant dihedral angle in six simple initial compounds of the shape H_3_C-YH_*n*_ (Y = C, N, O, Si, P, and S), *N*-methylacetamide (as a prototype of the peptide bond), and five peptide-capped amino acids (Gly, Ala, Val, Ile, and Leu) because of the protein direction of FFLUX. In order to test the reliability of the chosen level of theory (B3LYP/IGLO-III), the ^3^*J*(H,H′) values of ethane, methylamine, and methanol were calculated and confirmed to be close to the experiment. Furthermore, nicely fitted Karplus equations of ^3^*J*(H,H′) emerged for all 12 compounds in the whole range of the relevant dihedral angle. Because the Fermi contact term is typically the dominant contribution to ^3^*J*(H,H), similar excellent correlations are found except for Ala, Val, and Leu. However, when values are separated in two groups, depending on the dihedral angle (|θ| > 90° and |θ| < 90°), their correlations also become excellent (*R*^2^ > 0.999).

Finally, we observed that, for NMA and all five amino acids, the correlations between ^3^*J*(H,H′) and V_xc_*R* (where *R* is the internuclear distance) are always stronger than those with DI. Looking at all 12 systems, correlations between ^3^*J*(H,H′) and V_xc_ can be excellent (*R*^2^ > 0.91) but only for five systems. In conclusion, except for methanol, the inter-hydrogen exchange-correlation energy V_xc_(H,H′) makes the best contact with the experiment, through ^3^*J*(H,H′), when multiplied with the internuclear distance *R*_HH_′. However, the three types of plot, ^3^*J*(H,H′) against DI, V_xc_, or V_xc_*R*, each indicate that the fit improves if two lines are fitted instead of one, based on the value of the dihedral angle, |θ| > 90° or |θ| < 90°.

Looking at the future, such experimental reassurance may boost the newly proposed Relative Energy Gradient (REG) method,^57^ which computes chemical insight. For example, REG resolved^58^ the controversy surrounding the origin of the planar torsional energy barrier in biphenyl. The knowledge that the interatomic exchange-correlation energy has a link with experiment bolsters REG’s findings. For example, if REG ranks the exchange-correlation energy of the O···H hydrogen bond interaction in a classic water dimer configuration as five times weaker than its electrostatic counterpart, then the current work may lend this finding credibility. After all, *J*-couplings have been known to be associated with purely covalent bonds since the 1950s, but four decades later, direct evidence emerged^59^ for the presence of *J*-couplings between magnetically active nuclei on both sides of the hydrogen bond.

## Supporting Information Available

Table S1. Average value of ^3^*J*(H,H′) (Hz) calculated at the B3LYP computational level with eight different basis sets; Table S2. ^3^*J*(H,H′) (Hz), DI, V_xc_ (kJ/mol) for H_3_C-YH_*n*_, Y = C, N, O, Si, P, and S and *N*-methylacetamide (NMA) as a function of the dihedral angle (°) H–C–Y–H or H^N^–N–C^α^–H^α^ (NMA); Table S3. Fitted Karplus equations for ^3^*J*(H,H′); Table S4. Linear relationship between ^3^*J*(H,H′) and the Fermi contact (FC) term (Hz); Table S5. Karplus-like relationships between DI and the dihedral angle, θ; Figure S1. ^3^*J*(H,H′) as a function of DI and V_xc_; and Figure S2. FC term (Hz) as a function of DI and V_xc_ (PDF)
