# Supplementary material for: Linking the Interatomic Exchange-Correlation Energy to Experimental J-Coupling Constants
Source: J Phys Chem A. 2023 Jan 6;127(2):468–76. doi: 10.1021/acs.jpca.2c07693 (PMC9869393; doi:10.1021/acs.jpca.2c07693)
Supplement: Supplementary file 1 — jp2c07693_si_001.pdf [file jp2c07693_si_001.pdf]

Supporting Information

Linking the interatomic exchange-correlation energy to experimental J-coupling constants

Ibon Alkorta[a] and Paul L.A. Popelier[b]

[a] Instituto de Química Médica (CSIC), Juan de la Cierva, 3, 28006-Madrid, Spain

[b] Department of Chemistry, University of Manchester
Oxford Road, Manchester M13 9PL, Great Britain

Index

Pg. S2 Table S1. Average value of $^3J(H,H')$ (Hz) calculated at B3LYP computational level with 8 different basis sets.

Pg. S3-S6 Table S2. $^3J(H,H')$ (Hz), DI, V_{xc} (kJ/mol) for H_3C-YH_n, Y = C, N, O, Si, P and S and *N*-methylacetamide (NMA) as a function of dihedral angle (°) H-C-Y-H or H^N-N-C^α-H^α (NMA).

Pg. S7 Table S3. Fitted Karplus equations for $^3J(H,H')$.

Pg. S8 Table S4. Linear relationship between $^3J(H,H')$ and the Fermi Contact (FC) term (Hz).

Pg. S9 Table S5. Karplus-like relationships between DI and the dihedral angle, θ.

Pg. S10-S14 Figure S1. $^3J(H,H')$ as a function of DI and V_{xc}.

Pg. S15-S19 Figure S2. FC term (Hz) as a function of DI and V_{xc}.

Table S1. Average value of $^{3}J(H,H')$ (Hz) calculated at B3LYP level with 8 different basis sets. The B3LYP/6-311++G(d,p) optimised geometry has been used in all cases, except were indicated.

Basis set	CH_3CH_3	CH_3NH_2	CH_3OH
6-311++G(d,p)	7.13[e]	6.28	5.37
pcJ2	8.87	7.82	6.59
pcJ3	8.96		
pcJ4	8.95		
cc-pVTZ	7.43		
ccJ-pVTZ	8.76		
IGLO-III	7.88		
IGLO-III[a]	7.88	7.00	5.82
Experiment	7.992-8.005[b]	7.0[c]	5.535[d]

[a] Geometry optimized at B3LYP/IGLO-III level.
[b] Taken from Table 5 in Kaski, J.; Lantto, P.; Vaara, J.; Jokisaari, J. *J. Am. Chem. Soc.* **1998,** *120*, 3993-4005.
[c] Wielogórska, E.; Makulski, W.; Koźmiński, W.; Jackowski, K., *J. Mol. Struc.* **2004,** *704*, 305-309.
[d] Kupka, T., *Mag. Reson. Chem.* **2009,** *47*, 674-683; measured in diluted $CDCl_3$.
[e] All 8 calculated values are actually an average of the values at -60°, +60° and 180°.

Table S2. $^3J(H,H')$ (Hz), DI, V_{xc} (kJ/mol) for H_3C-YH_n, Y=C, N, O, Si, P and S, and NMA as a function of dihedral angle (°) H-C-Y-H or H^N-N-C^α-H^α (NMA).

CH_3-CH_3

J	DI	V_{xc}	θ
4.22	0.0052	-0.50	120.0
13.33	0.0105	-1.74	0.0
8.12	0.0082	-0.78	135.0
1.42	0.0029	-0.29	-105.0
12.49	0.0100	-1.64	15.0
12.08	0.0111	-1.06	150.0
0.41	0.0019	-0.21	-90.0
10.20	0.0084	-1.37	30.0
15.01	0.0133	-1.27	165.0
1.33	0.0023	-0.32	-75.0
7.03	0.0062	-0.99	45.0
16.08	0.0141	-1.34	-180.0
3.79	0.0039	-0.60	-60.0

CH_3-NH_2

J	DI	V_{xc}	θ
12.01	0.0066	-1.08	0.0
4.16	0.0045	-0.45	120.2
3.70	0.0045	-0.44	-119.9
3.99	0.0044	-0.44	-119.5
12.02	0.0066	-1.08	0.7
3.85	0.0046	-0.44	120.5
10.90	0.0062	-0.98	15.0
8.49	0.0069	-0.69	136.3
0.68	0.0025	-0.24	-105.2
0.55	0.0025	-0.25	-102.4
11.25	0.0063	-1.06	18.9
8.49	0.0073	-0.71	137.4
8.29	0.0051	-0.79	30.0
12.50	0.0093	-0.92	151.9
-0.31	0.0014	-0.16	-90.5
-0.60	0.0017	-0.18	-86.4
8.71	0.0052	-0.85	35.5
12.76	0.0097	-0.95	153.1
5.06	0.0038	-0.55	45.0
15.23	0.0109	-1.08	166.9
0.62	0.0015	-0.20	-76.0
0.26	0.0019	-0.24	-71.9
5.54	0.0036	-0.58	50.0
15.39	0.0111	-1.09	167.1

2.08	0.0026	-0.35	59.6
16.18	0.0115	-1.13	-178.8
2.74	0.0023	-0.35	-62.0
2.08	0.0026	-0.35	-59.6
2.74	0.0023	-0.35	62.0
16.18	0.0115	-1.13	178.8

CH_3-OH

J	DI	V_{xc}	θ
10.03	0.0044	-0.75	0.0
3.55	0.0034	-0.35	-120.4
9.26	0.0042	-0.70	15.0
7.29	0.0052	-0.53	135.0
0.63	0.0019	-0.20	-105.9
7.16	0.0035	-0.57	30.0
10.91	0.0070	-0.72	149.5
-0.80	0.0011	-0.12	-91.5
4.37	0.0025	-0.40	45.0
13.58	0.0084	-0.85	164.1
-0.54	0.0010	-0.13	-77.2
14.66	0.0090	-0.91	-180.0
1.40	0.0015	-0.23	61.4

CH_3-SiH_3

J	DI	V_{xc}	θ
3.04	0.0064	-0.56	-120.0
6.65	0.0092	-1.52	0.0
6.01	0.0087	-0.76	135.0
0.72	0.0044	-0.40	-105.0
6.14	0.0087	-1.44	15.0
8.89	0.0110	-0.95	150.0
-0.43	0.0032	-0.33	-90.0
4.73	0.0075	-1.21	30.0
10.96	0.0126	-1.08	165.0
-0.27	0.0031	-0.40	-75.0
2.83	0.0058	-0.90	45.0
11.71	0.0131	-1.12	-180.0
0.97	0.0041	-0.60	-60.0

CH_3-PH_2

J	DI	V_{xc}	θ
14.62	0.0129	-2.27	0.0
5.33	0.0078	-0.72	121.8
2.23	0.0056	-0.52	-119.8
0.00	0.0037	-0.39	-93.0
12.16	0.0120	-2.11	28.7
10.02	0.0112	-1.01	147.1
13.08	0.0115	-2.00	15.0
9.76	0.0109	-1.00	137.3
-0.25	0.0036	-0.36	-105.3
0.18	0.0034	-0.43	-78.0
8.70	0.0096	-1.63	44.3
13.92	0.0139	-1.25	161.7
10.12	0.0094	-1.60	30.0
13.60	0.0136	-1.23	152.3
-0.64	0.0030	-0.35	-90.9
2.31	0.0045	-0.66	-63.3
4.87	0.0067	-1.08	58.9
16.18	0.0155	-1.39	175.8
6.54	0.0071	-1.14	45.0
15.94	0.0153	-1.38	166.8
0.90	0.0038	-0.55	-76.6
5.59	0.0064	-1.03	-48.9
1.57	0.0043	-0.64	72.9
16.27	0.0155	-1.40	-170.5
6.07	0.0067	-1.08	47.0
16.12	0.0154	-1.39	168.6
1.22	0.0041	-0.59	-74.7
6.07	0.0067	-1.08	-47.0
1.22	0.0041	-0.59	74.7
16.12	0.0154	-1.39	-168.6

CH_3-SH

J	DI	V_{xc}	θ
14.30	0.0105	-1.92	0.0
3.38	0.0055	-0.52	119.7
3.38	0.0055	-0.52	-119.7
13.34	0.0099	-1.78	15.0
7.21	0.0079	-0.73	134.0
0.59	0.0037	-0.35	-105.6
10.74	0.0082	-1.42	30.0
11.11	0.0104	-0.95	148.5
-0.50	0.0026	-0.27	-91.7
7.22	0.0060	-0.98	45.0

14.13	0.0123	-1.13	163.2
0.31	0.0026	-0.32	-77.9
3.23	0.0038	-0.56	61.8
15.57	0.0133	-1.22	179.8
3.13	0.0038	-0.55	-62.2

NMA, $^3J(H^N,H^\alpha)$ (see Figure 2 of the main text for θ)

J	DI	V_{xc}	θ
7.02	0.0042	-0.69	0.0
3.18	0.0035	-0.34	120.1
3.19	0.0035	-0.34	-120.1
6.57	0.0039	-0.64	15.0
6.03	0.0051	-0.50	134.7
1.02	0.0022	-0.22	-105.5
5.30	0.0033	-0.52	30.0
9.06	0.0067	-0.66	149.5
-0.07	0.0014	-0.14	-90.9
3.41	0.0023	-0.35	45.0
11.47	0.0079	-0.78	164.4
0.06	0.0011	-0.13	-76.2
1.47	0.0015	-0.21	60.0
12.46	0.0084	-0.82	179.3
1.31	0.0015	-0.20	-61.4
2.62	0.0032	-0.31	-116.8
6.98	0.0042	-0.68	3.3
3.77	0.0038	-0.37	123.3

Table S3. Fitted Karplus equations for $^{3}J(H,H')$.

$^{3}J(H,H')$ (Y=C, N, O, Si, P and S) (θ = H-C-Y-H')

$^{3}J(A,B) = C_2\cos^2\theta + C_1\cos\theta + C_0$

System	Fitted equation [x = cosθ]	R^2
CH_3-CH_3	J(Hz) = $14.284x^2 - 1.0733x + 0.4261$	0.998
CH_3-NH_2	J(Hz) = $14.5x^2 - 1.7879x - 0.3693$	0.993
CH_3-OH	J(Hz) = $13.154x^2 - 2.1843x - 0.7832$	0.999
CH_3-SiH_3	J(Hz) = $9.6012x^2 - 2.3862x - 0.4025$	0.999
CH_3-PH_2	J(Hz) = $15.783x^2 - 0.7757x - 0.2306$	0.982
CH_3-SH	J(Hz) = $15.307x^2 - 0.3895x - 0.3679$	0.998

$^{3}J(H^N,H^\alpha)$ ($\theta = H^N$-N-C^α-H^α)

System	Fitted equation [x=cosθ]	R^2	# points
NMA	J(Hz) = $9.8281x^2 - 2.3215x - 0.1863$	0.994	18
Gly (Scan[a])	J(Hz) = $8.5613x^2 - 0.7641x - 0.5661$	0.992	48[b]
Gly (Scan + Min)	J(Hz) = $10.509x^2 - 1.886x - 0.2522$	0.984	66
Ala (Scan)	J(Hz) = $7.5076x^2 - 0.1352x + 0.0698$	0.958	24
Ala (Scan + Min)	J(Hz) = $7.6629x^2 - 0.1713x + 0.0504$	0.957	35
Val (Scan)	J(Hz) = $7.9409x^2 - 0.469x + 0.0659$	0.983	24
Val (Scan + Min)	J(Hz) = $8.2194x^2 - 0.267x - 0.0363$	0.956	40
Ile (Scan)	J(Hz) = $9.4669x^2 - 1.5637x + 0.2519$	0.995	24
Ile (Scan + Min)	J(Hz) = $9.4753x^2 - 1.3365x + 0.3018$	0.963	49
Leu (Scan)	J(Hz) = $7.9372x^2 - 0.1851x - 0.0056$	0.961	24
Leu (Scan + Min)	J(Hz) = $7.7506x^2 - 0.5028x + 0.0699$	0.931	52
All-AA+NMA (Scan + Min)	J(Hz) = $8.9416x^2 - 0.9864x - 0.0378$	0.880	259

[a] "Scan" refers to the energy profile controlled by the φ dihedral angle as described in the section on computational details of the main text.

[b] There are 48 points in this fit instead of the 24 of the other 4 amino acids because both α hydrogen atoms are included instead of just one.

Table S4. Linear relationship between $^3J(H,H)$ and the FC term (Hz).

System	Fitted equation	R^2
CH_3-CH_3	J(Hz) = 1.01 FC - 0.09	0.9998
CH_3-NH_2	J(Hz) = 1.02 FC - 0.10	0.9996
CH_3-OH	J(Hz) = 1.03 FC - 0.12	0.999
CH_3-SiH_3	J(Hz) = 0.995 FC - 0.09	0.9995
CH_3-PH_2	J(Hz) = 0.998 FC - 0.07	0.999
CH_3-SH	J(Hz) = 1.02 FC - 0.20	0.998
NMA	J (Hz) = 1.02 FC - 0.08	0.999
Gly (Scan + Min)	J (Hz) = 1.01 FC - 0.04	0.999
Ala (Scan + Min)	J (Hz) = 0.97 FC - 0.07	0.943*
Val (Scan + Min)	J (Hz) = 0.89 FC + 0.43	0.940*
Ile (Scan + Min)	J (Hz) = 1.00 FC + 0.08	0.999
Leu (Scan + Min)	J (Hz) = 0.94 FC + 0.03	0.938*

*The fits of these systems show excellent correlations if the values are separated based on the dihedral angles as shown just below.

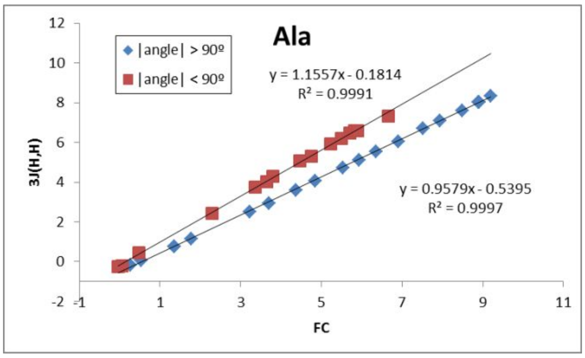

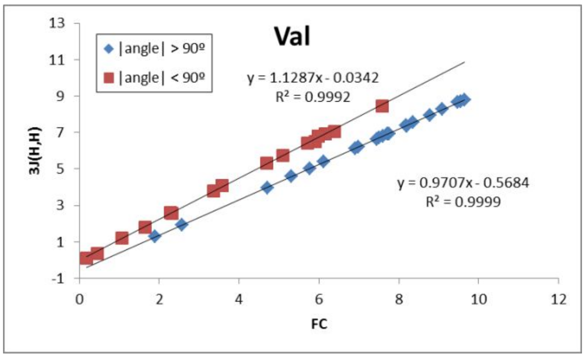

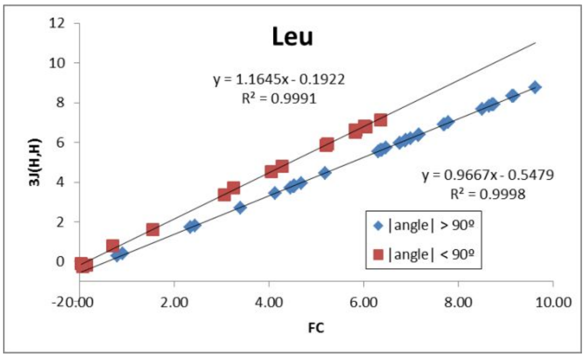

Table S5. Karplus-like relationships between DI and the dihedral angle, θ, where θ = H^N-N-C^α-H^α or H-C-Y-H.

System	Fitted equation [x = cos (θ)]	R^2
CH_3-CH_3	DI = 0.0104x^2 - 0.0016x + 0.0019	0.999
CH_3-NH_2	DI = 0.0075x^2 - 0.0022x + 0.0016	0.996
CH_3-OH	DI = 0.0056x^2 - 0.0021x + 0.0011	0.997
CH_3-SiH_3	DI = 0.008x^2 - 0.002x + 0.0032	0.9995
CH_3-PH_2	DI = 0.0109x^2 - 0.0014x + 0.0033	0.974
CH_3-SH	DI = 0.0093x^2 - 0.0014x + 0.0025	0.9995

System	Fitted equation [x = cos (θ)]	R^2
NMA	DI = 0.005x^2 - 0.002x + 0.0013	0.9995
Gly (Scan + Min)	DI = 0.0054x^2 - 0.0018x + 0.0011	0.990
Ala (Scan + Min)	DI = 0.0054x^2 - 0.0014x + 0.0013	0.983
Val (Scan + Min)	DI = 0.0058x^2 - 0.0015x + 0.0011	0.988
Ile (Scan + Min)	DI = 0.0056x^2 - 0.0015x + 0.0011	0.977
Leu (Scan + Min)	DI = 0.0052x^2 - 0.0015x + 0.0012	0.988
All-AA and NMA	DI = 0.0055x^2 - 0.0016x + 0.0011	0.981

Figure S1. ${}^3J(H,H')$ as a function of DI, V_{xc} and $V_{xc}R$. The data have been separated based on the dihedral angle values.

CH_3-CH_3

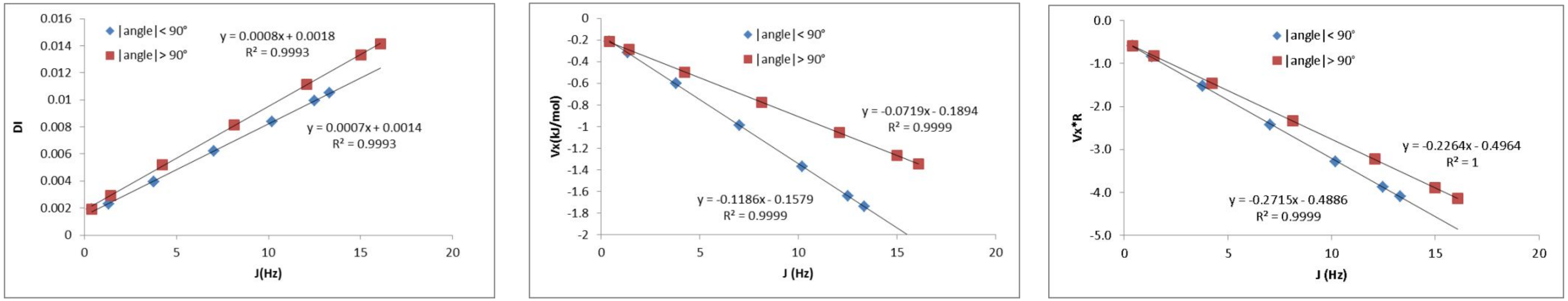

CH_3-NH_2

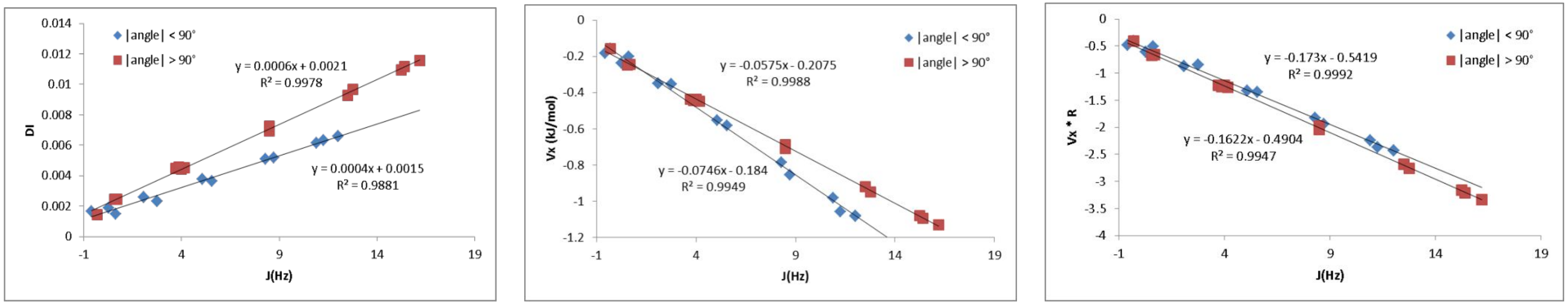

CH_3-OH

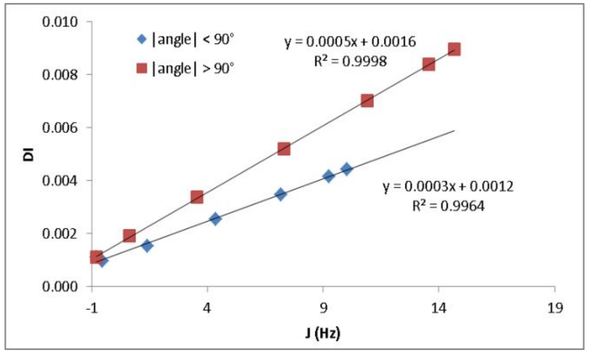

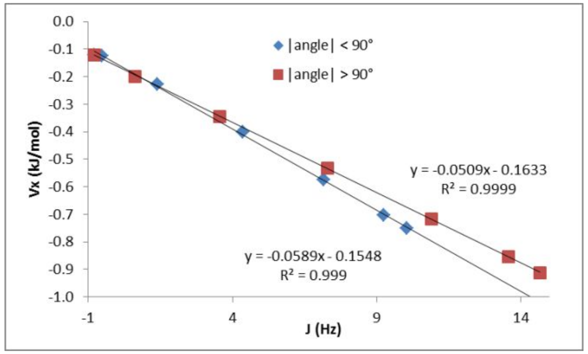

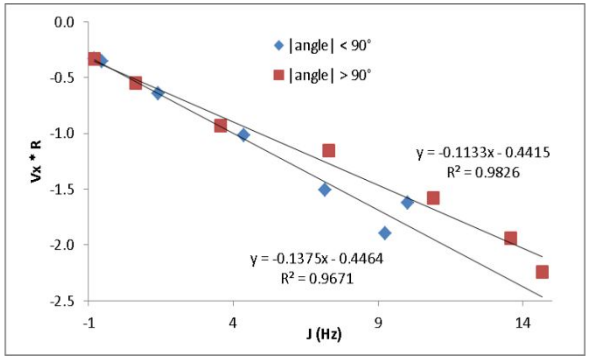

CH_3-SiH_3

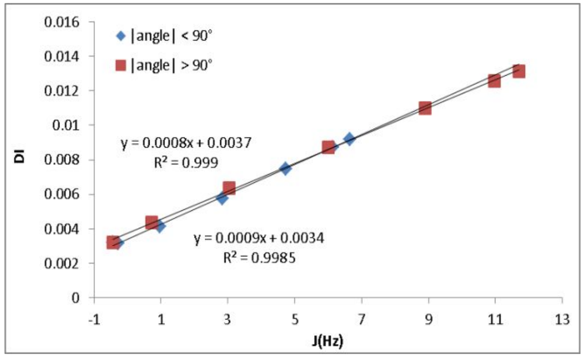

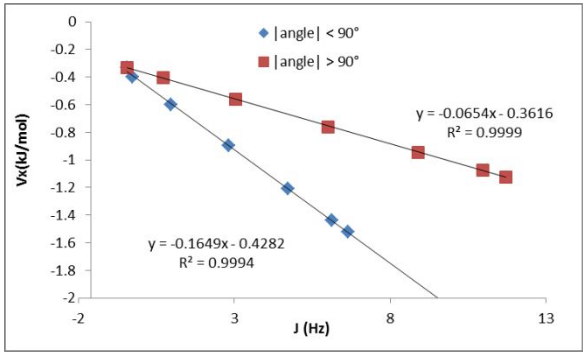

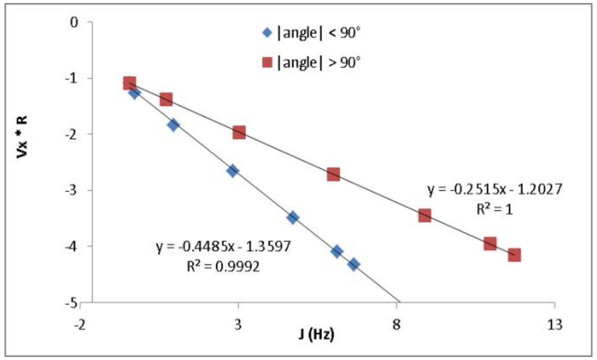

CH_3-PH_2

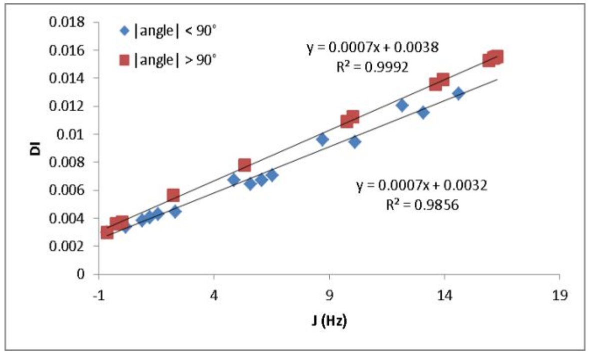

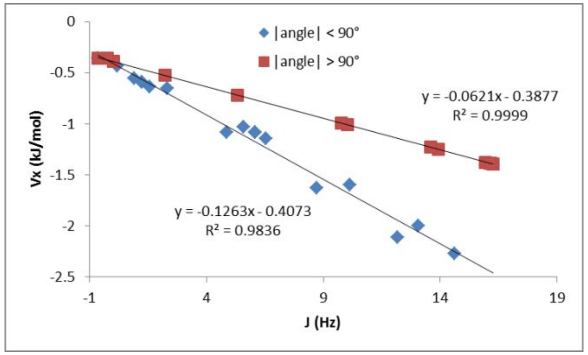

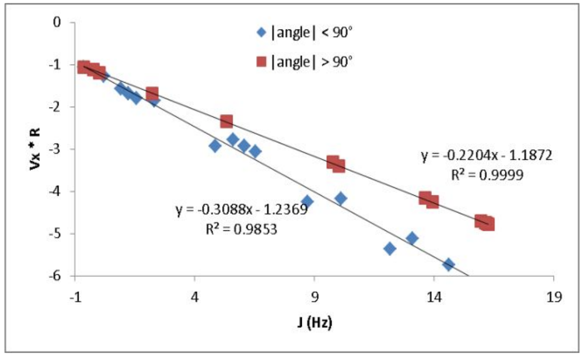

CH_3-SH

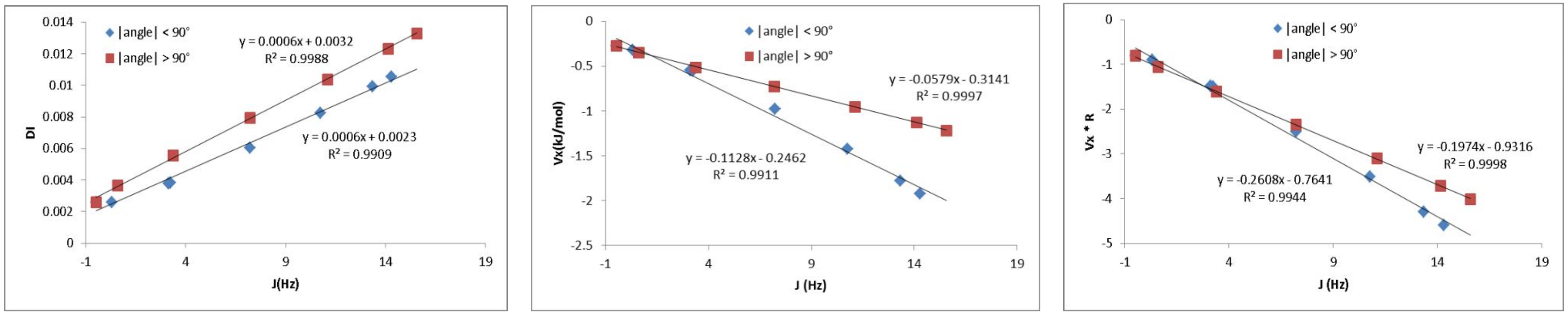

NMA

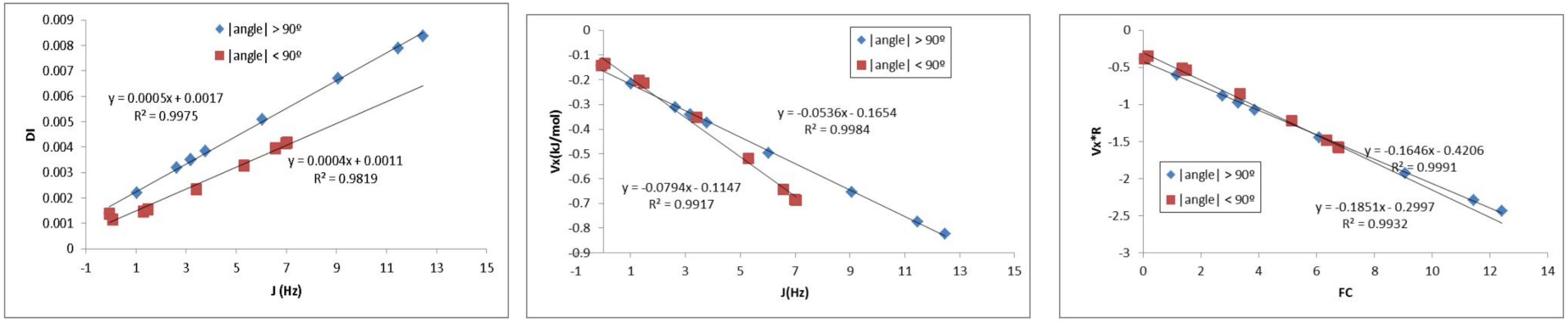

Gly

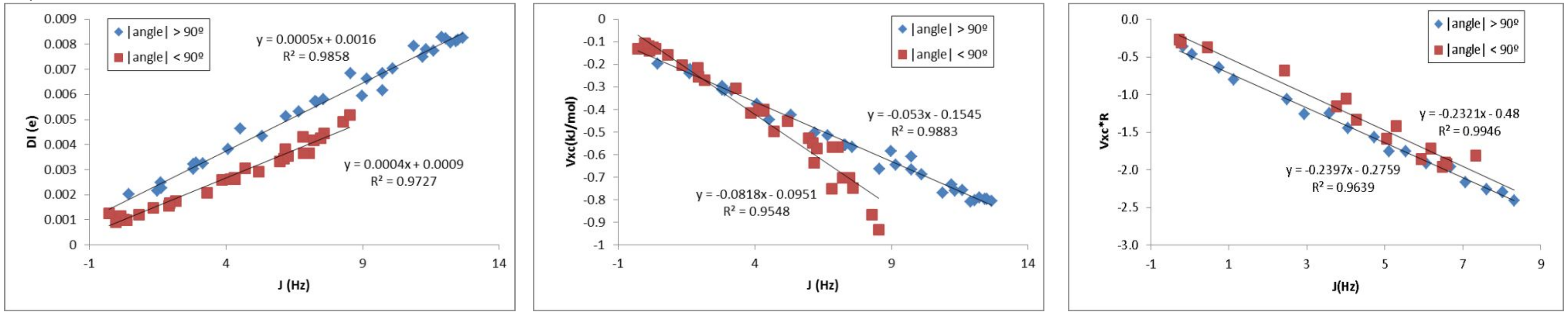

Ala

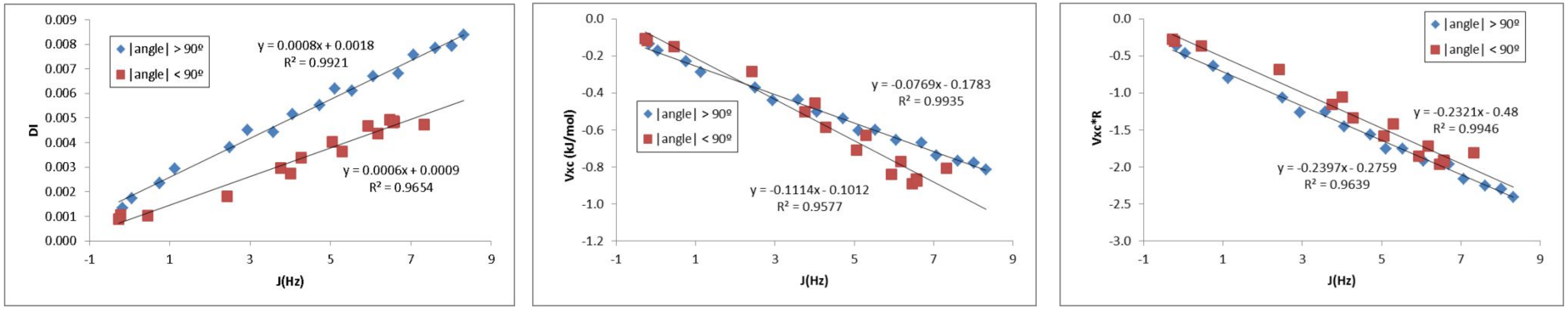
|angle| > 90º
|angle| < 90º
y = 0.0008x + 0.0018
R² = 0.9921
y = 0.0006x + 0.0009
R² = 0.9654
DI
J(Hz)
y = -0.0769x - 0.1783
R² = 0.9935
y = -0.1114x - 0.1012
R² = 0.9577
Vxc (kJ/mol)
J(Hz)
y = -0.2321x - 0.48
R² = 0.9946
y = -0.2397x - 0.2759
R² = 0.9639
Vxc*R
J(Hz)

Val

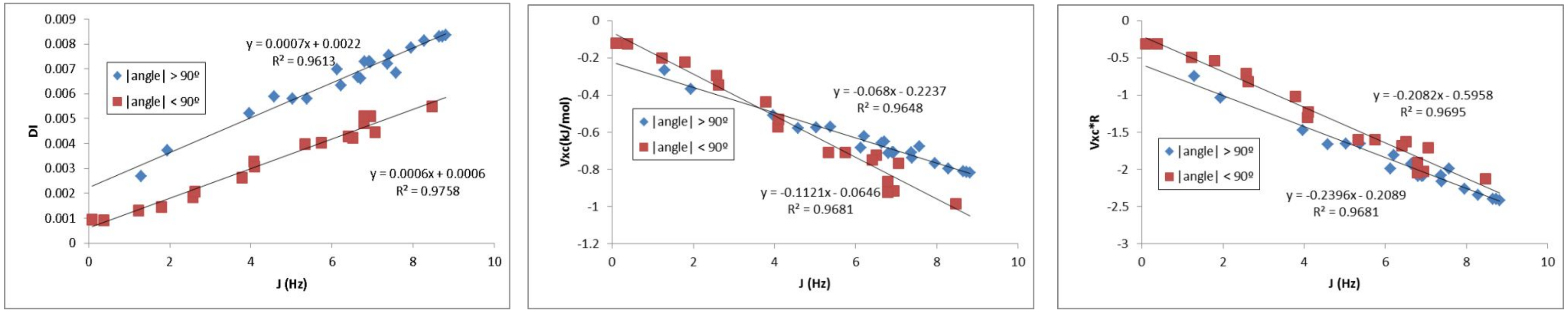
|angle| > 90º
|angle| < 90º
y = 0.0007x + 0.0022
R² = 0.9613
y = 0.0006x + 0.0006
R² = 0.9758
DI
J (Hz)
y = -0.068x - 0.2237
R² = 0.9648
y = -0.1121x - 0.0646
R² = 0.9681
Vxc(kJ/mol)
J (Hz)
y = -0.2082x - 0.5958
R² = 0.9695
y = -0.2396x - 0.2089
R² = 0.9681
Vxc*R
J (Hz)

Ile

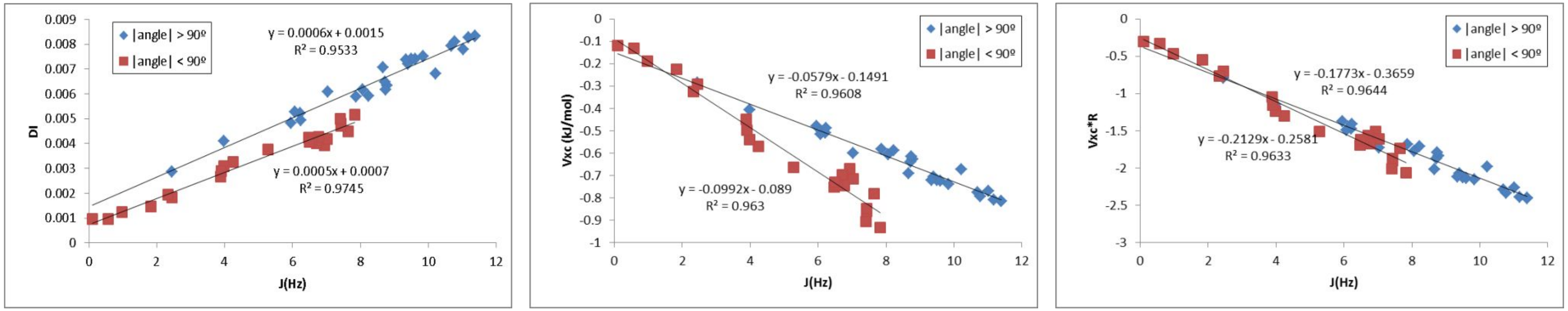
|angle| > 90º
|angle| < 90º
y = 0.0006x + 0.0015
R² = 0.9533
y = 0.0005x + 0.0007
R² = 0.9745
DI
J(Hz)
y = -0.0579x - 0.1491
R² = 0.9608
y = -0.0992x - 0.089
R² = 0.963
Vxc (kJ/mol)
J(Hz)
y = -0.1773x - 0.3659
R² = 0.9644
y = -0.2129x - 0.2581
R² = 0.9633
Vxc*R
J(Hz)

Leu

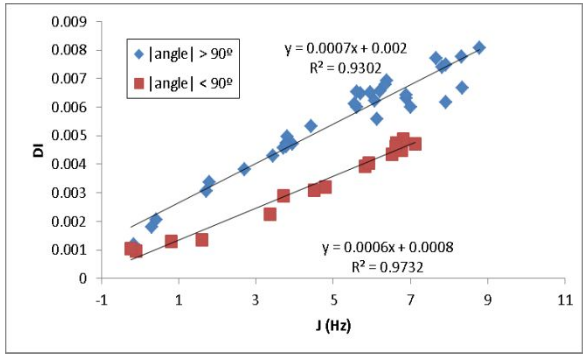

DI
J (Hz)
|angle| > 90º
|angle| < 90º
y = 0.0007x + 0.002
R² = 0.9302
y = 0.0006x + 0.0008
R² = 0.9732

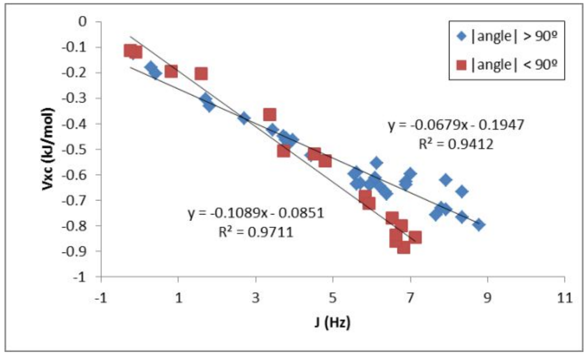

Vxc (kJ/mol)
J (Hz)
|angle| > 90º
|angle| < 90º
y = -0.0679x - 0.1947
R² = 0.9412
y = -0.1089x - 0.0851
R² = 0.9711

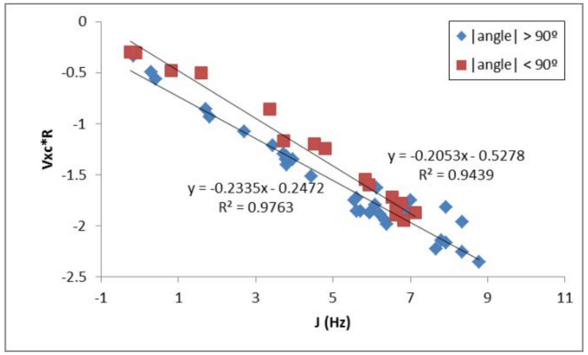

Vxc*R
J (Hz)
|angle| > 90º
|angle| < 90º
y = -0.2053x - 0.5278
R² = 0.9439
y = -0.2335x - 0.2472
R² = 0.9763

Figure S2. FC term (Hz) as a function of DI, V_{xc} and $V_{xc}R$. The data have been separated based on the dihedral angle value.

CH_3-CH_3

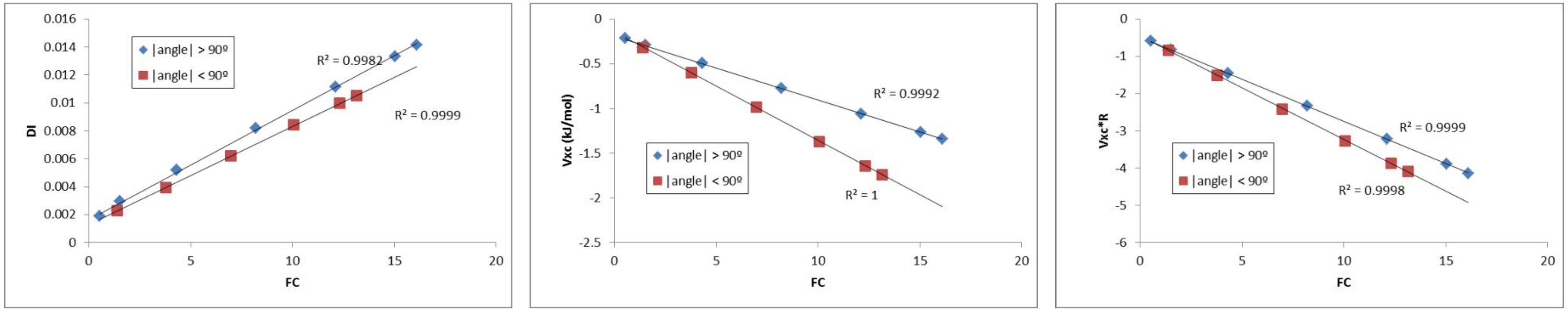

CH_3-NH_2

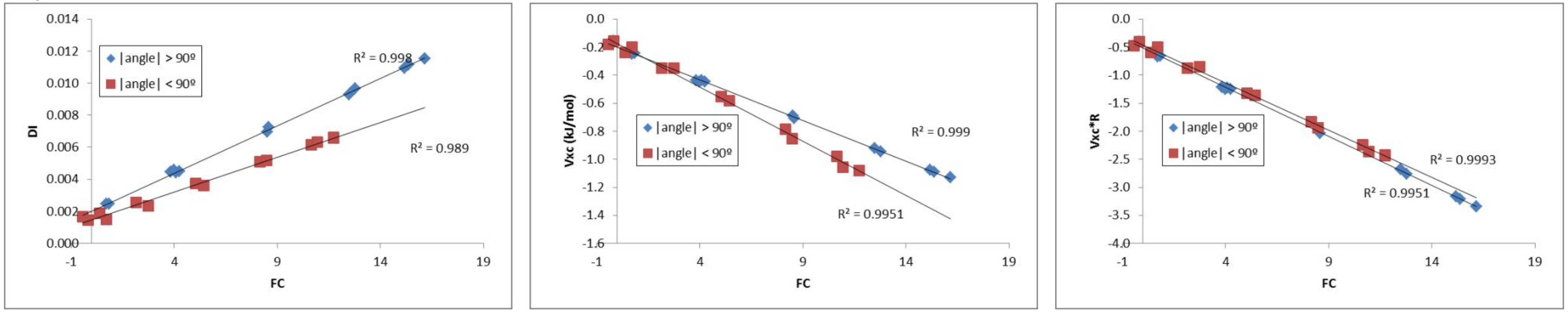

CH_3-OH

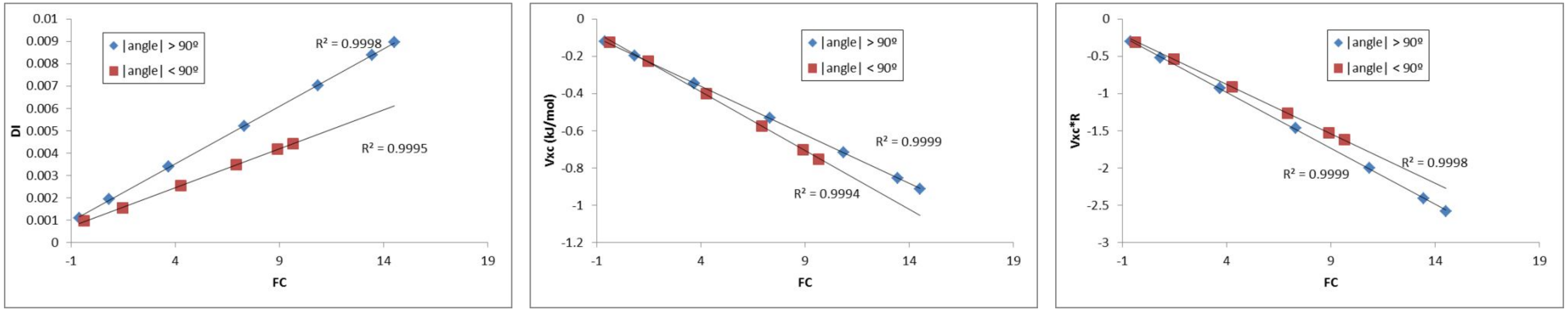

CH_3-SiH_3

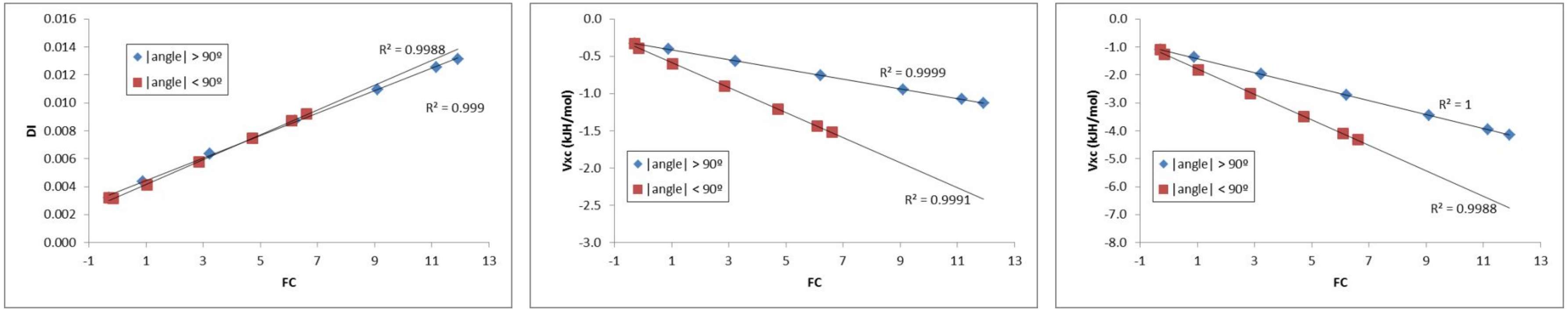

CH_3-PH_2

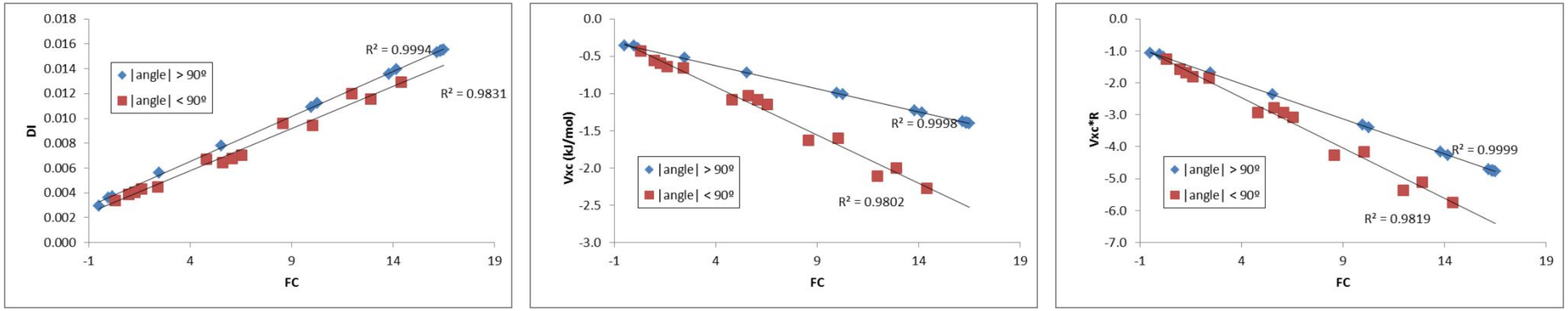

CH_3-SH

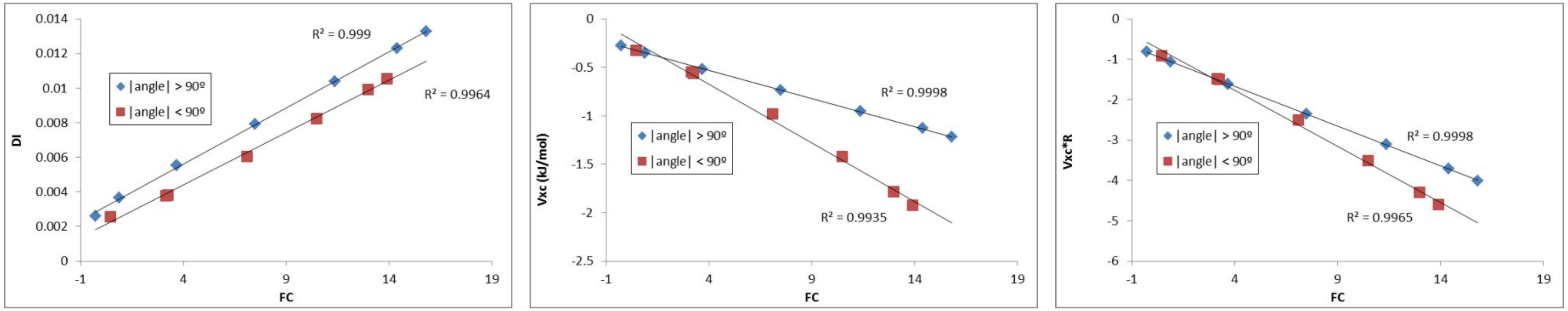

NMA

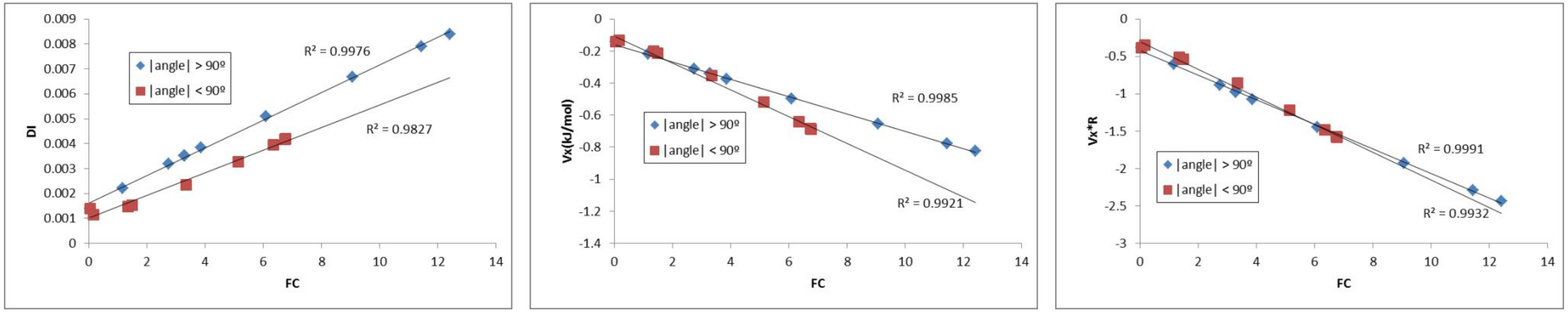

Gly (Scan + Min)

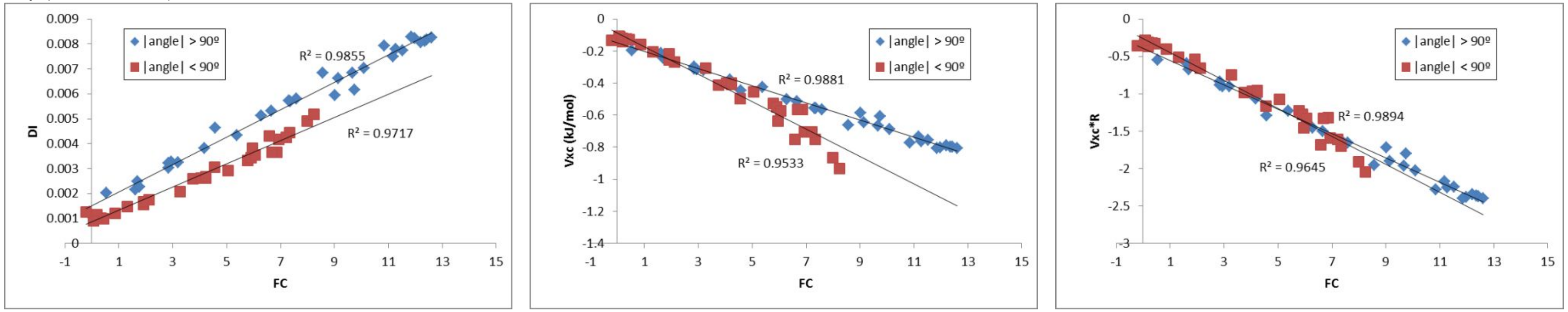

Ala (Scan + Min)

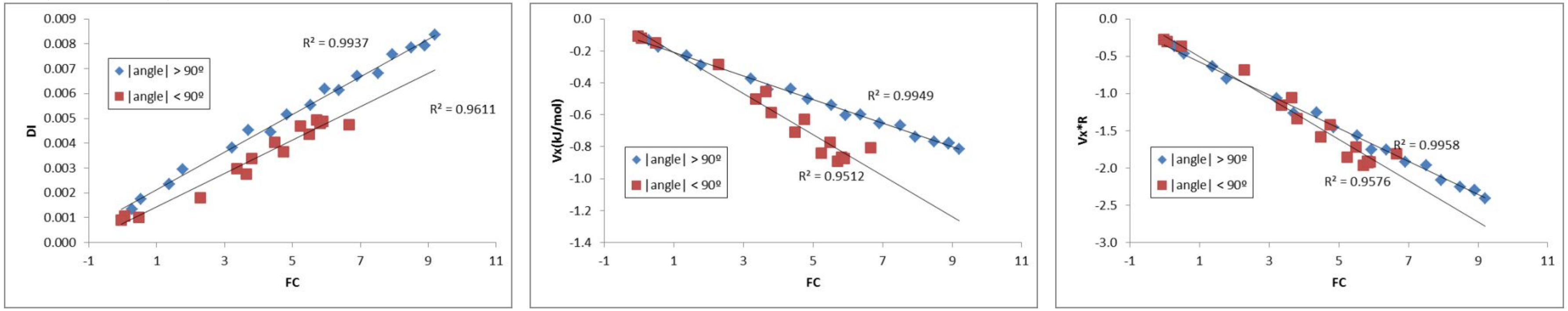

Val (Scan + Min)

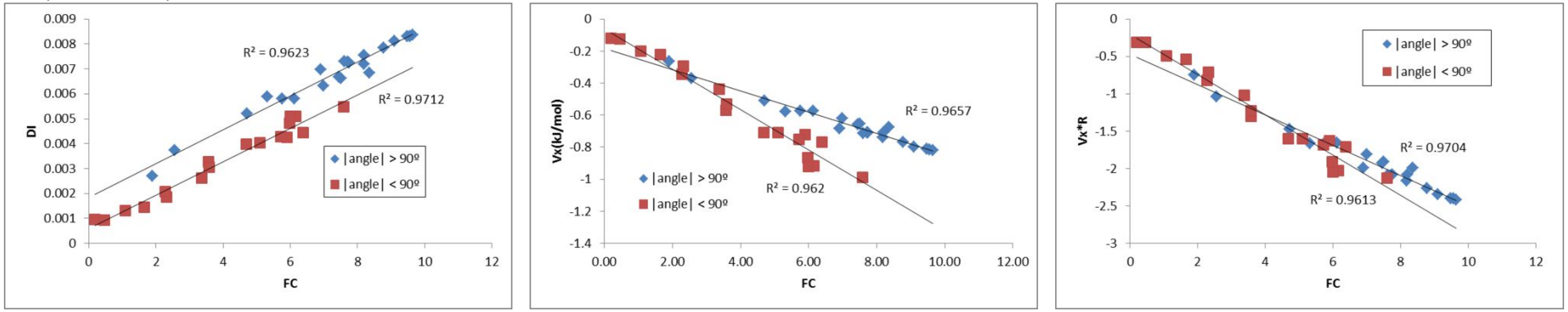

Ile (Scan + Min)

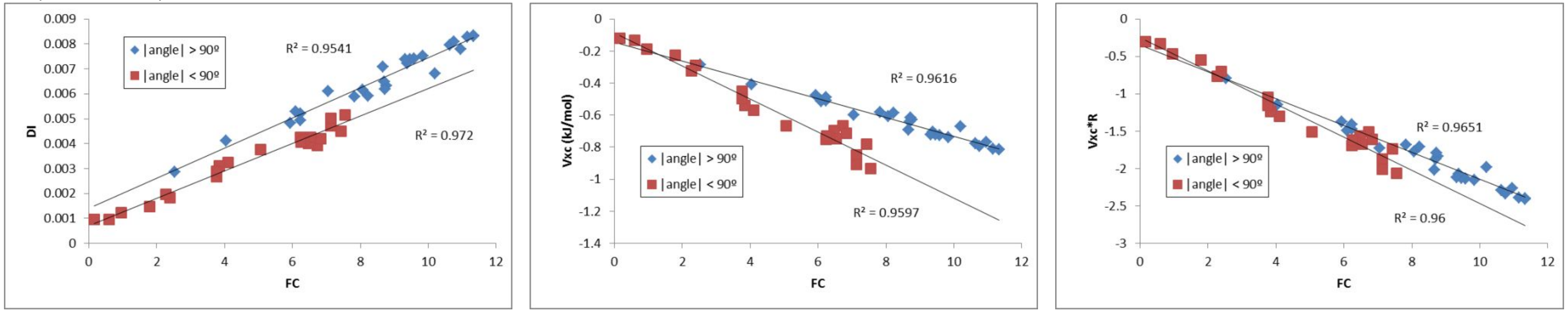

Leu (Scan + Min)

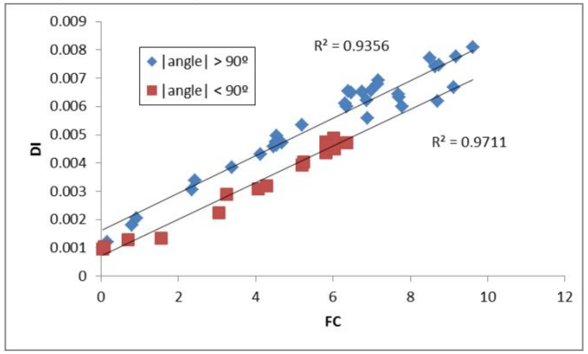

DI
FC
|angle| > 90º
|angle| < 90º
R² = 0.9356
R² = 0.9711

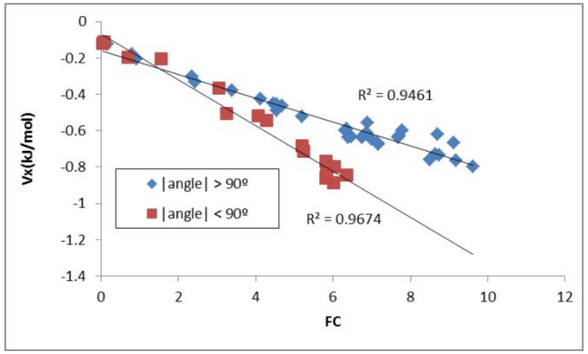

Vx(kJ/mol)
FC
|angle| > 90º
|angle| < 90º
R² = 0.9461
R² = 0.9674

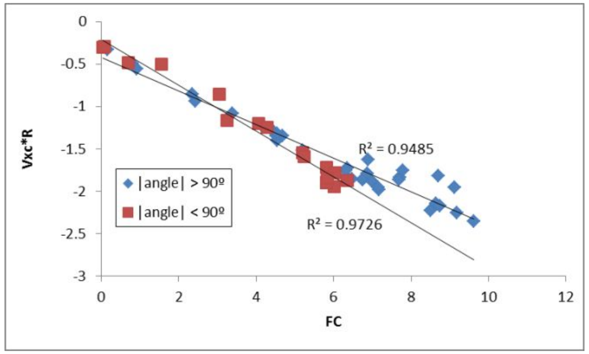

Vxc*R
FC
|angle| > 90º
|angle| < 90º
R² = 0.9485
R² = 0.9726
